# Endoscopic resection with an over-the-scope clip for recurrence of gastric cancer on the esophagus adjacent to the anastomosis after total gastrectomy

**DOI:** 10.1055/a-2362-0979

**Published:** 2024-07-29

**Authors:** Taro Iwatsubo, Yuichiro Hatano, Kentaro Matsuo, Ryo Tanaka, Kazuhiro Ota, Sang-Woong Lee, Hiroki Nishikawa

**Affiliations:** 1 13010 Second Department of Internal Medicine, Osaka Medical and Pharmaceutical University, Takatsuki, Japan; 2 38588 Endoscopy Center, Osaka Medical and Pharmaceutical University Hospital, Takatsuki, Japan; 3 13010 Department of Pathology, Faculty of Medicine, Osaka Medical and Pharmaceutical University, Takatsuki, Japan; 4 13010 Department of General and Gastroenterological Surgery, Osaka Medical and Pharmaceutical University, Takatsuki, Japan

The endoscopic resection of recurrent lesions on the postoperative anastomosis is challenging. Endoscopic resection with an over-the-scope (OTS) clip has been reported to be useful for subepithelial and fibrotic lesions 1 2 . Herein, we report a case of endoscopic resection for the recurrence of gastric cancer adjacent to the anastomosis after total gastrectomy using an OTS clip.

A man in his sixties underwent total gastrectomy after neoadjuvant chemotherapy for advanced gastric cancer 3 years prior, and the pathological result was a mixture of mucinous, poorly differentiated, and signet-ring cell adenocarcinoma (ypT3, ypN2, and ycM0). Surveillance endoscopy revealed a flat, elevated lesion with erosions, 8 mm in size, adjacent to the anastomosis of the esophagus and small intestine. The lesion resembled a subepithelial tumor covered with normal epithelium ( Fig. 1 ). The biopsied specimen from this lesion pathologically revealed poorly differentiated and signet-ring cell adenocarcinoma, which was suspected to be a residue or implantation of gastric cancer after gastrectomy. Therefore, a deep-layer resection was required to achieve complete resection. However, conventional endoscopic mucosal resection or submucosal dissection was thought to be significantly difficult because of severe fibrosis of the anastomosis and subepithelial lesions.

**Fig. 1:**
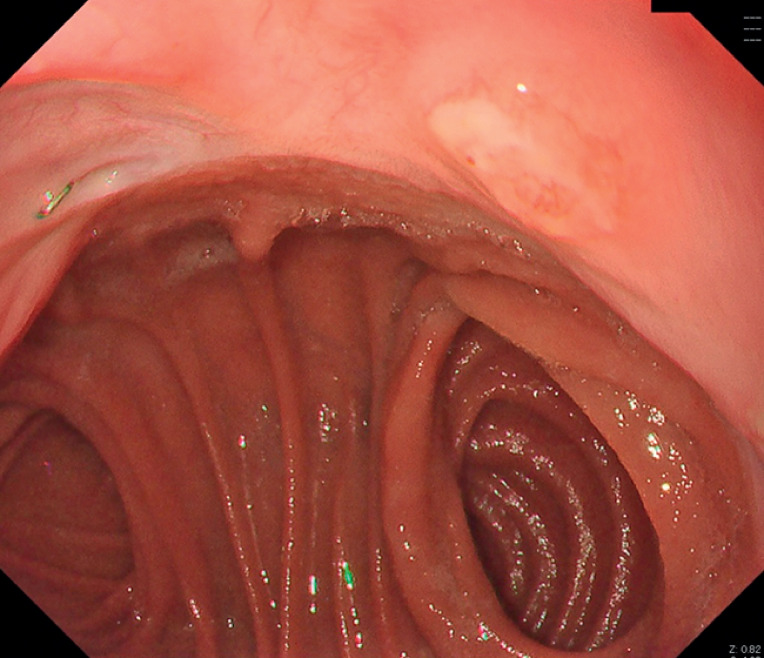
A subepithelial tumor covered with normal epithelium is located on the esophagus on the oral side of the anastomosis.

We performed endoscopic resection using an OTS clip system (Ovesco Endoscopy GmbH, Tübingen, Germany) to easily and safely resect the recurrent lesion at the deeper layer ( Video 1 ). Markings were placed outside the lesion at the snare tip. The 11-mm version of the OTS clip system was subsequently deployed at the base of the lesion, and the lesion was resected above the OTS clip using a 10-mm snare (Captivator II; Boston Scientific, Marlborough, Massachusetts, USA) ( Fig. 2 , Fig. 3 , Fig. 4 ). The procedure took 15 min. The lesion was successfully resected without complications ( Fig. 5 ). This method was effective for such a small recurrent lesion adjacent to the anastomosis.

Endoscopic resection with an over-the-scope clip for recurrent gastric cancer on the esophagus adjacent to the anastomosis after total gastrectomy.Video 1

**Fig. 2:**
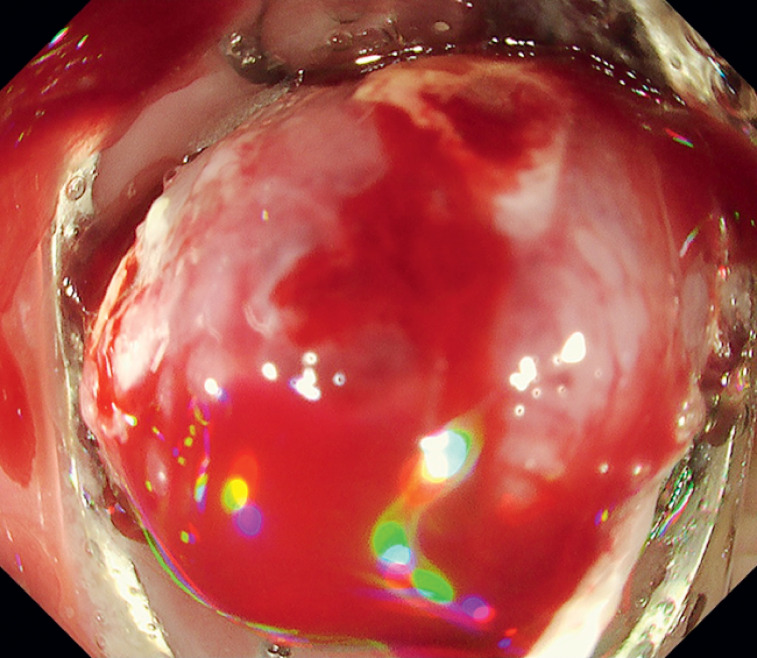
The lesion is aspirated to make sure that the markings around the lesion are within the attachment, and the over-the-scope clip is fired.

**Fig. 3:**
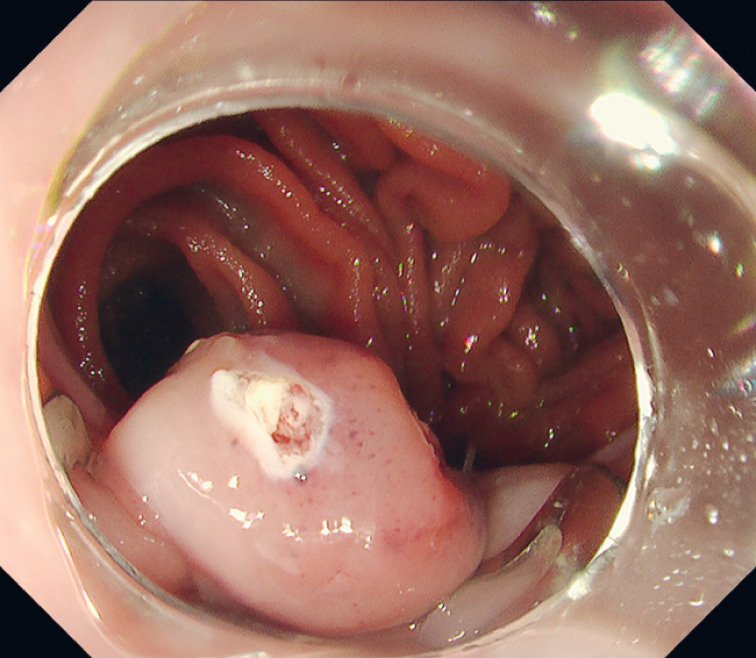
A pseudo-protruding polyp is formed above the over-the-scope clip.

**Fig. 4:**
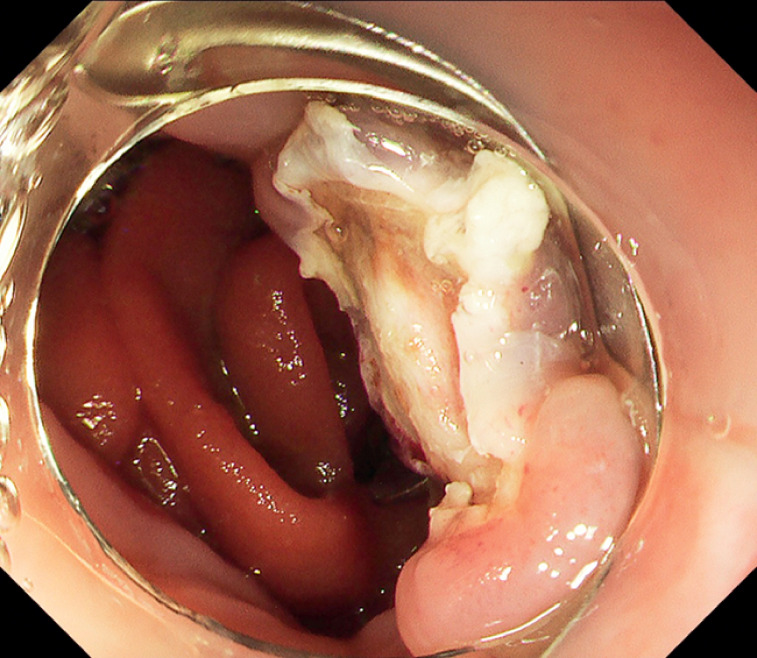
A pseudo-protruding polyp is formed above the over-the-scope clip.

**Fig. 5:**
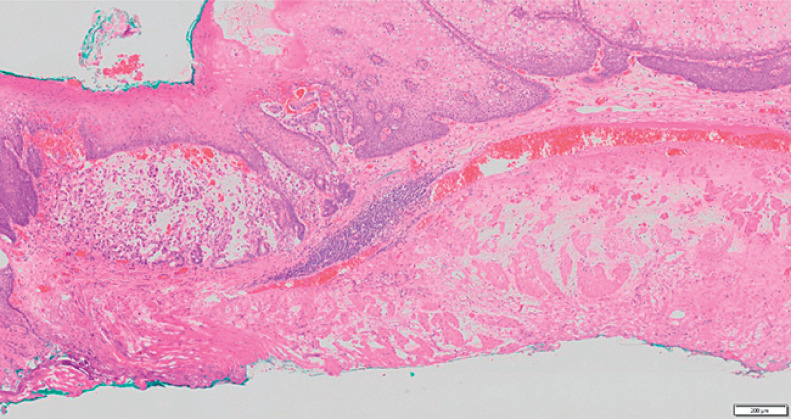
The tumor is located predominantly in the lamina propria mucosae and successfully completely resected with a negative margin.

Endoscopy_UCTN_Code_TTT_1AO_2AG_3AF

## Notes

Endoscopy E-Videos https://eref.thieme.de/e-videos
*E-Videos* is an open access online section of the journal *Endoscopy* , reporting on interesting cases and new techniques in gastroenterological endoscopy. All papers include a high-quality video and are published with a Creative Commons CC-BY license. Endoscopy E-Videos qualify for HINARI discounts and waivers and eligibility is automatically checked during the submission process. We grant 100% waivers to articles whose corresponding authors are based in Group A countries and 50% waivers to those who are based in Group B countries as classified by Research4Life (see: https://www.research4life.org/access/eligibility/ ). This section has its own submission website at https://mc.manuscriptcentral.com/e-videos .
